# Anti-Viral Photodynamic Inactivation of T4-like Bacteriophage as a Mammalian Virus Model in Blood

**DOI:** 10.3390/ijms231911548

**Published:** 2022-09-30

**Authors:** Patrícia Santos, Ana T. P. C. Gomes, Leandro M. O. Lourenço, Maria A. F. Faustino, Maria G. P. M. S. Neves, Adelaide Almeida

**Affiliations:** 1 CESAM, Department of Biology, University of Aveiro, 3810-193 Aveiro, Portugal; 2 LAQV–REQUIMTE, Department of Chemistry, University of Aveiro, 3810-193 Aveiro, Portugal

**Keywords:** antimicrobial photodynamic therapy, viral inactivation, blood, plasma, photosensitizers, porphyrins, methylene blue

## Abstract

The laboratorial available methods applied in plasma disinfection can induce damage in other blood components. Antimicrobial photodynamic therapy (aPDT) represents a promising approach and is approved for plasma and platelet disinfection using non-porphyrinic photosensitizers (PSs), such as methylene blue (MB). In this study, the photodynamic action of three cationic porphyrins (Tri-Py(+)-Me, Tetra-Py(+)-Me and Tetra-S-Py(+)-Me) towards viruses was evaluated under white light irradiation at an irradiance of 25 and 150 mW·cm^−2,^ and the results were compared with the efficacy of the approved MB. None of the PSs caused hemolysis at the isotonic conditions, using a T4-like phage as a model of mammalian viruses. All porphyrins were more effective than MB in the photoinactivation of the T4-like phage in plasma. Moreover, the most efficient PS promoted a moderate inactivation rate of the T4-like phage in whole blood. Nevertheless, these porphyrins, such as MB, can be considered promising and safe PSs to photoinactivate viruses in blood plasma.

## 1. Introduction

Blood plays vital functions for organism maintenance and survival, participating in the transport of oxygen, among other important substances, pathogen identification/elimination, and wound healing, among other functions [1,2].

Whole blood transfusions were common until the 1970s, but currently, blood derivative products (red blood cells, platelets, and plasma) are more used, according to the needs of each patient [1,3,4]. In accordance with the Food and Drug Administration (FDA), transfusion transmissible infections (TTIs) can be defined as infections caused by a pathogen that is potentially transmissible through the blood supply and causes severe damage in the organism [5]. To ensure the quality and safety of transfusion, haemovigilance systems have been implemented in many countries [6]. Screening tests for the presence of Human Immunodeficiency Virus (HIV), Hepatitis B (HBV), and Hepatitis C (HCV) viruses and the *Treponema pallidum* bacterium are recommended for all blood donations [7,8].

Despite the safety of donor blood products being the main concern of the medical community, the emerging infectious diseases remain a threat to the blood supply [5,8]. Many infectious agents can be transmitted through transfusion, such as viruses, bacteria, fungi, protozoa, or other parasites [8,9]. The window period where pathogenic agents are present but not detectable intensifies this problem [2,10]. Therefore, there is a consensus that the disinfection of blood and derivatives merits special attention. The conventional disinfection techniques approved to ensure transfusion safety are mainly intended for plasma (especially against viruses). Despite being regulated, these techniques can damage cellular fractions, such as platelets and erythrocytes [1,7]. Moreover, these treatments do not have the same efficacy against all microorganisms; for instance, enveloped lipidic viruses are more easily inactivated than non-enveloped lipidic viruses [8,11].

Light-based therapeutic approaches are used for the reduction of pathogens in blood. Ultraviolet (UV) light is essentially applied in plasma and platelet disinfection. This type of irradiation causes damage to the microbial genetic material, preventing its replication; however, this technique produces free radicals that are extremely cytotoxic [8,12]. Antimicrobial Photodynamic Therapy (aPDT) is also used for blood disinfection. This therapeutic approach involves the use of a photosensitizer (PS) that when excited by visible light in the presence of dioxygen produces reactive oxygen species (ROS) such as singlet oxygen (^1^O_2_) and/or hydroxyl radicals (OH^•^), superoxide (O_2_^•−^), and hydrogen peroxide (H_2_O_2_), responsible for significant changes in nearby biological molecules and for the consequent cell death [13]. One of the major advantages of this method is its multitarget approach affecting mainly outer structures, such as lipids and proteins [14,15]. Consequently, the development of resistance to this method is highly improbable [16,17]. aPDT can act on bacteria, viruses, fungi, and other parasites [15,18]. Nevertheless, the effectiveness of aPDT relies on the type of microorganisms [19,20]. For instance, aPDT is considered more effective against Gram-positive bacteria than Gram-negative bacteria due to dissimilarities in the membrane permeability [14,21]. Regarding viruses, enveloped viruses are inactivated more easily than non-enveloped viruses [14,22].

With respect to the application of aPDT to the reduction of pathogens in blood products, there are only three PSs approved for plasma and platelets treatment: amotosalen (a psolaren), riboflavin (or vitamin B_2_), and MB. The use of amotosalen and riboflavin requires the use of UVA and UVB, respectively, which may lead to the formation of harmful free radicals that can affect plasma proteins and platelets. On the other hand, the use of MB combined with visible light (instead of UV light) may be an advantage for pathogen inactivation in plasma [1,23]. MB has already shown efficacy in the aPDT of several enveloped viruses, such as HIV or West Nile virus [24,25] and the protozoan *Trypanosoma cruzi* [26]. Regarding non-enveloped viruses, the effectiveness is slightly lower, and although it is not effective against Hepatitis A virus, it was efficient in the photoinactivation of the non-enveloped *Parvovirus B19* [24,27]. The adverse effects of MB have been evaluated over the last years and some doubts have arisen about MB efficacy in the treatment of plasma in patients suffering from thrombotic thrombocytopenic purpura [23,28,29]. Although it is approved for plasma disinfection in several European countries, in France it was removed from the market [24,25] due to allergic reactions detected in a few patients who received plasma treated with MB [23]. Previous studies on the MB-mediated aPDT against bloodborne viruses such as HIV-1 also evidenced the damage to red blood cells and platelets, which is still a limiting aspect during viral disinfection of blood [30].

Even though there is no approved treatment for red blood cells or whole blood, the efficacy of aPDT mediated by several PSs in blood disinfection was reported [31,32]. The major difficulty in the development of an aPDT protocol for erythrocytes or whole blood stems is the sensitivity of these cells to light. Red blood cells are sensitive to ROS once some of nonselective cation channels in the erythrocyte membrane are activated by oxidative stress, promoting a cascade of events responsible for an alteration in membrane stability and a sodium influx, with the resultant erythrocytes swelling, which might cause haemolysis of red blood cells [33]. However, it has been shown by our research group that aPDT with tetrapyrrolic PSs is a promising technique to photoinactivate bacteria [31] and fungi [32], not only in plasma but also in the whole blood, without causing significant hemolysis in the red blood cells [31,32]. Despite the fact that this methodology has been studied in the aPDT of bacteria and fungi, there are no reports on the use of tetrapyrrolic macrocycles for the photoinactivation of viruses in blood plasma and/or whole blood.

**Figure 1:**
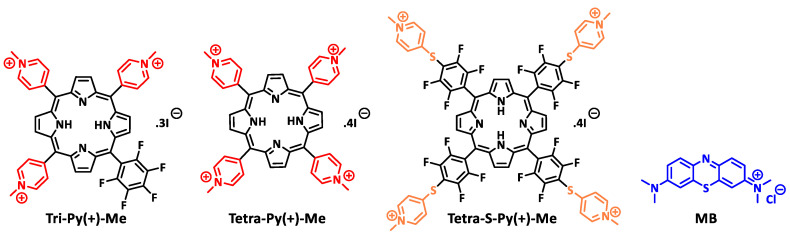
Chemical structures of the cationic porphyrins and of methylene blue (MB).

Therefore, the aim of this work was to contribute to the existing literature a new class of PSs for the disinfection of blood and blood plasma with efficacy towards a broad range of pathogens, including viruses. For this, the effectiveness of the three cationic porphyrins Tri-Py(+)-Me, Tetra-Py(+)-Me, and Tetra-S-Py(+)-Me (Figure 1) was evaluated to photoinactivate viruses in plasma and whole blood, and their efficiency was compared with MB efficacy. As viruses are important contaminants of blood products and the effectiveness of aPDT depends on the microorganisms’ type, for the development of an aPDT protocol with new PSs, it is necessary to test their effectiveness against viruses.

## 2. Results

The establishment of the best aPDT protocol to photoinactivate viruses in plasma and whole blood in the presence of Tri-Py(+)-Me, Tetra-Py(+)-Me, and Tetra-S-Py(+)-Me (Figure 1) required the previous evaluation of aPDT conditions that did not promote significant erythrocytes haemolysis.

### 2.1. Evaluation of aPDT Effect on Erythrocyte Osmotic Fragility

**Figure 2:**
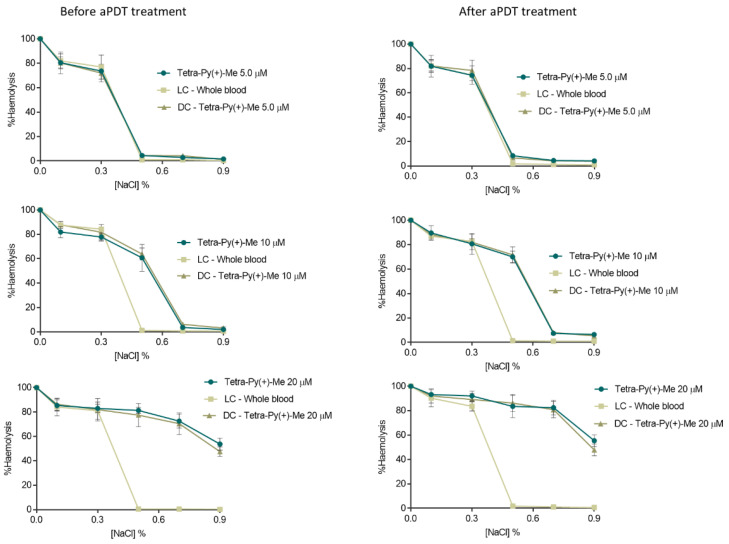
Erythrocyte osmotic fragility before and after aPDT treatment with Tetra-Py(+)-Me (5.0, 10 and 20 µM) and under white light irradiation at an irradiance of 150 mW·cm^−2^. All values represent the average of three independent assays. Error bars indicate the standard deviation and in some cases are collapsed with the symbols. LC—Light control (whole blood under light); DC—Dark control (whole blood incubated with PS without light).

**Figure 3:**
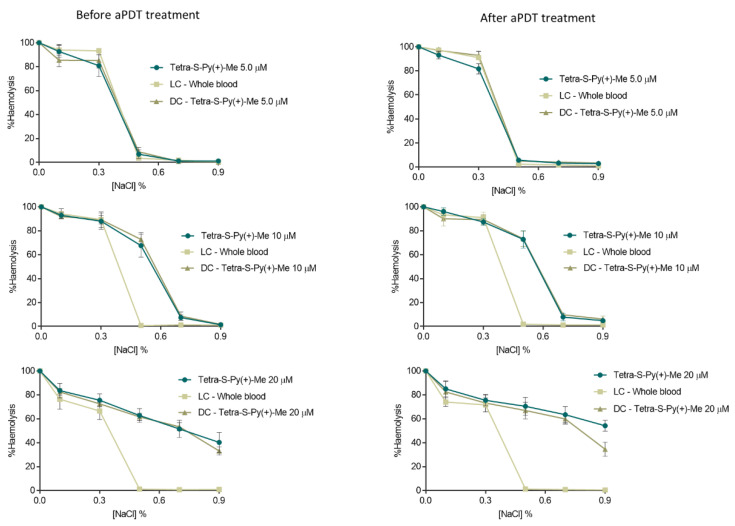
Erythrocyte osmotic fragility before and after aPDT treatment with Tetra-S-Py(+)-Me (5.0, 10 and 20 µM) and under white light irradiation at an irradiance of 150 mW·cm^−2^. All values represent the average of three independent assays. Error bars indicate the standard deviation and in some cases are collapsed with the symbols. LC—Light control (whole blood under light); DC—Dark control (whole blood incubated with PS without light).

To select the safe aPDT conditions, assays concerning the erythrocyte osmotic fragility were performed for all PSs (Figure 1) at the same concentration used in the inactivation assays (10 µM) and also at a lower and a higher PS concentration, 5 and 20 µM, in whole blood before and after aPDT protocol (using white light at an irradiance of 150 mW·cm^−2^) [31,32]. Erythrocyte samples were added to tubes containing increasing concentrations of sodium chloride (NaCl) solution (0, 0.1, 0.3, 0.5, 0.7, and 0.9%) at pH 7.4 and the hemoglobin content on supernatant was spectrophotometrically quantified [31,32]. The results of the erythrocyte osmotic fragility in the presence of Tri-Py(+)-Me and MB were already reported in the same tested conditions [31,32]. Both PSs did not promote significant erythrocytes haemolysis after aPDT at 5.0 and 10 μM (ANOVA, *p* > 0.05) using the non-stress (isotonic) condition (0.9% NaCl, corresponding to the concentration of the blood). However, at 20 µM of Tri-Py(+)-Me, lysis of erythrocytes occurred even for the isotonic conditions [31,32], a fact that was not observed for MB at the same concentration [32]. The osmotic fragility results obtained in the presence of Tetra-Py(+)-Me (Figure 2) and Tetra-S-Py(+)-Me (Figure 3) at 5.0 µM show no significant haemolysis (ANOVA, *p* > 0.05) between the sample and the light and dark controls for 0.5–0.9% NaCl conditions, proving that these PSs are not toxic to erythrocytes at this concentration. At 10 µM, no significant haemolysis (ANOVA, *p* > 0.05) was also observed for these PSs for 0.7 and 0.9% NaCl when compared with controls. However, at 0.5% NaCl, a significant erythrocytes haemolysis (ANOVA, *p* < 0.05) was observed for these PSs when compared with the light control at the same NaCl concentration, being 60% and 70% for Tetra-Py(+)-Me and 67% and 73% for Tetra-S-Py(+)-Me, before and after aPDT treatment, respectively. The highest tested concentration (20 µM) haemolysis rate of 53% and 55% was detected for Tetra-Py(+)-Me (ANOVA, *p* < 0.05) and 40% e 54% for Tetra-S-Py(+)-Me (ANOVA, *p* < 0.05) before and after treatment, respectively, in isotonic conditions (0.9% NaCl). In fact, earlier studies have also reported that higher concentrations of porphyrins affected the erythrocyte membrane [34,35], which seems to indicate that the osmotic stress is caused by the high PS concentration and not by the aPDT treatment and/or the ROS presence [31]. Moreover, the fact that these porphyrins at the highest concentrations promote high haemolysis rates, unlike what happens with MB, can also be due to the presence of the dimethyl sulfoxide (DMSO) used to prepare the stock solutions of porphyrinic PSs, whereas the MB stock solution was prepared in phosphate-buffered saline (PBS). In fact, previous studies reported that erythrocyte osmotic fragility can occur at percentages of DMSO of 4% [32], even under isotonic conditions (0.9% NaCl), which corresponds to the amount of DMSO present at a concentration of 20 µM of PS; lower percentages of DMSO (1% and 2%), which coincide with the percentage present at PS concentrations of 5 and 10 µM, did not promote significant haemolysis in 0.7% and 0.9% NaCl solutions before and after aPDT protocol [32].

Nevertheless, it is important to highlight that no significant haemolysis (ANOVA, *p* > 0.05), was observed for the three porphyrinic PSs at concentrations of 5.0 and 10 μM under the isotonic conditions, which means that these concentrations are safe to be used in blood and blood products disinfection. Since plasma and whole blood are complex matrices with a large amount of organic matter that may require higher PS concentrations for an effective photoinactivation rate, a PS concentration of 10 µM was used in the aPDT assays against T4-like phage.

### 2.2. Photodynamic Inactivation of T4-Like Phage in PBS, Plasma, and Whole Blood

The photodynamic efficacy of Tri-Py(+)-Me, Tetra-Py(+)-Me, Tetra-S-Py(+)-Me, and MB towards viruses particles was first compared in PBS. As previously stated, this study was conducted with a T4-like phage used as a model of mammalian viruses.

**Figure 4:**
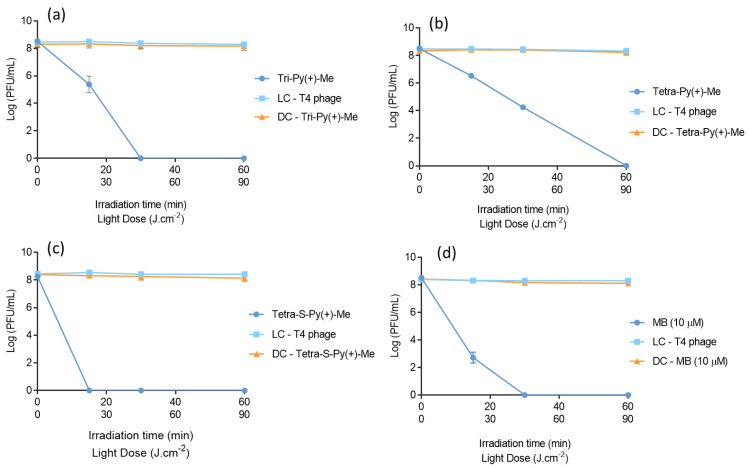
T4-like phage photoinactivation in PBS in the presence of (**a**) Tri-Py(+)-Me, (**b**) Tetra-Py(+)-Me, (**c**) Tetra-S-Py(+)-Me, and (**d**) MB at a concentration of 10 µM and under white light irradiation at an irradiance of 25 mW·cm^−2^. All values represent the average of three independent assays in duplicate. Error bars indicate the standard deviation and in some cases are collapsed with the symbols. Lines were used to connect the data points. LC—Light control; DC—Dark control.

The aPDT assays were performed with PSs at the established safe concentration of 10 μM and irradiated with white light at an irradiance of 25 mW·cm^−2^ for 60 min. The obtained results are summarized in Figure 4 and show that all PSs efficiently inactivated the T4-like phage up to the detection limit of the method, although with different inactivation profiles. Tetra-S-Py(+)-Me was the most effective PS (ANOVA, *p* < 0.05), promoting a fast decrease in the phage concentration to the detection limit of the method after 15 min of light irradiation (Figure 4c). Tri-Py(+)-Me and MB attained this limit after 30 min of irradiation with white light (Figure 4a,d). Tetra-Py(+)-Me was the least effective PS in PBS, since the photoinactivation of T4-like phage until the detection limit was reached only after 60 min of irradiation (Figure 4b). Light and dark controls demonstrated no significant decreases in the T4-like phage concentration (ANOVA, *p* > 0.05). These results suggest that the viability of this bacteriophage was affected neither by the light irradiation in the PS absence (light control) nor by the presence of PS without light irradiation (dark control).

The photodynamic efficiency of all PSs against the T4-like phage was also assessed in plasma at 10 µM under white light irradiation. Considering the high complexity of the biological matrix in relation to PBS, it was necessary to increase the irradiation time to 270 min and the light irradiance to 150 mW·cm^−2^, both in plasma and in whole blood assays. Similar studies performed in our group were taken into account in the selection of these conditions to photoinactivate bacteria [31] and fungi [32].

**Figure 5:**
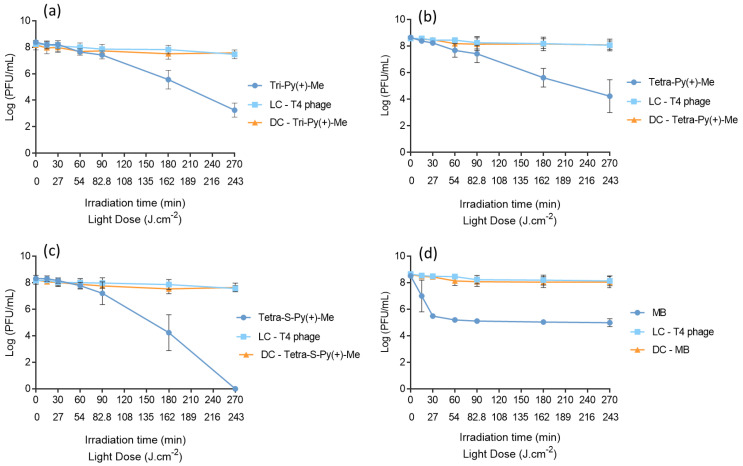
T4-like phage photoinactivation in plasma in the presence of (**a**) Tri-Py(+)-Me, (**b**) Tetra-Py(+)-Me, (**c**) Tetra-S-Py(+)-Me, and (**d**) MB at 10 µM and under white light irradiation at an irradiance of 150 mW·cm^−2^. All values represent the average of three independent assays in duplicate. Error bars indicate the standard deviation and in some cases are collapsed with the symbols. Lines were used to connect the data points. LC—Light control; DC—Dark control.

The obtained results ©n the plasma demonstrate significant differences when compared to the PBS assays (Figure 5). For the three porphyrins, T4-like phage photoinactivation was only observed after 90 min of light irradiation. After this irradiation period (180 and 270 min of irradiation), significant differences (ANOVA, *p* < 0.05) between the T4-like phage concentration in samples and controls were observed. As observed in Figure 4c, the Tetra-S-Py(+)-Me had promoted a decrease in the viability of the virus up to the detection limit of the method after 270 min of irradiation (corresponding to a reduction of 8 log_10_ PFU/mL (ANOVA, *p* < 0.05). A reduction of 5.1 log_10_ PFU/mL (ANOVA, *p* < 0.05) in the T4 phage survival was observed for Tri-Py(+)-Me (Figure 4a) and 4.4 log_10_ PFU/mL (ANOVA, *p* < 0.05) for Tetra-Py(+)-Me (Figure 4b) after 270 min of irradiation. With regards to MB, this approved PS revealed a different profile from the porphyrin derivatives. In this case, a reduction in phage viability of 1.5 log_10_ PFU/mL occurred after 15 min of irradiation (ANOVA, *p* < 0.05). After 30 min of irradiation, MB caused a decrease of 3 log_10_ PFU/mL (ANOVA, *p* < 0.05) in the phage concentration, and no further reduction was detected at the end of the irradiation period (270 min). All PSs demonstrated significant differences (ANOVA, *p* < 0.05) among themselves after 270 min of aPDT treatment (Figure 5a–d). Light and dark controls did not induce a decrease in the T4-like phage concentration (ANOVA, *p* > 0.05), which means that neither irradiation nor PS under dark conditions affects T4-like phage viability.

## 3. Discussion

Conventional disinfection techniques approved for plasma (e.g., wet and dry heat treatments) [7,10,28] proved to be efficient in the inactivation of human immunodeficiency virus (HIV) or hepatitis B virus (HBV) but are not effective against non-enveloped viruses and can cause damage to the plasma proteins [7]. Also, the solvent/detergent method (SD method) approved in some European countries and in the USA for plasma or protein concentrates disinfection [7,10], and effective against non-envelope viruses, has some limitations; the chemicals used promote negative effects in the erythrocyte membranes and platelets and need to be removed after treatment [1,36]. Other procedures used for blood plasma purification are based in chromatographic techniques using specific antibodies adsorbed and in physical methods to remove extracellular pathogens, such as nanofiltration or cell washing [1,8]. However, these techniques cannot be applied in concentrated platelets and erythrocytes, since cell membranes can bind non-specifically to the antibodies and intracellular pathogens are not filtrated or wash removed [37,38]. Moreover, other studies have reported using cationic photosensitizer-antibody conjugates against HIV env-expressing cells as photoimmunotherapy, in spite of these methods having some limitations [39,40].

Therefore, it is recognized that aPDT is a promising alternative for plasma disinfection, but the establishment of protocols with efficacy towards a broad range of pathogens including viruses are still necessary. Considering this, in this work the aPDT efficiency of three porphyrinic PSs, Tri-Py(+)-Me, Tetra-Py(+)-Me, and Tetra-S-Py(+)-Me, towards viruses in blood plasma and/or whole blood was evaluated for the first time. Since no significant haemolysis was observed for the three porphyrinic PSs at concentrations of 10 μM under isotonic conditions, all the aPDT assays were performed at this concentration, which is safe to be used in blood and blood products disinfection.

The study was conducted with an *Escherichia coli* T4-like bacteriophage, a ddDNA non-enveloped phage from the Caudovirales order, as a model of mammalian viruses [22,41]. This type of phage is commonly used as an indicator of the presence of enteric pathogens and has been used as a model of mammalian viruses in several photodynamic studies, showing that their inactivation mediated by aPDT is dependent on the number and position of the positive charges of the PSs, their hydrophobicity, light source, and total light dose. It is known that the photoinactivation of non-enveloped viruses occurs mainly by the interaction of ROS with the external proteins of the capsid, through degradation, cleavage, and cross-linking modifications, which leads to damages in fundamental structural and functional molecules. In the case of the T4-like phage, the degradation of several proteins was observed [42], including a long-tail fiber protein that is involved in the interaction with specific receptors on the cell host surface, previously reported by us [22].

Thus, it is notable that for all the PSs tested, the photoinactivation rate diminished in the plasma assays when compared to the PBS ones. In PBS, Tri-Py(+)-Me inactivated the T4-like phage up to the detection limit of the method (reduction of 8.0 log_10_ PFU/mL) after 30 min of irradiation (Figure 4a) and caused a T4-like phage inactivation of 5.1 log_10_ PFU/mL after 270 min of irradiation in plasma (Figure 5a). A similar profile was found for Tetra-Py(+)-Me; this PS inactivated the T4-like phage up to the detection limit of the method (reduction of 8.0 log_10_ PFU/mL) after 60 min of irradiation in PBS (Figure 4b) and caused a T4-like phage inactivation of 4.4 log_10_ PFU/mL after 270 min of irradiation in plasma (Figure 5b). Nevertheless, Tetra-S-Py(+)-Me was revealed to be the most effective PS, inactivating the T4-like phage up to the detection limit of the method not only in PBS (Figure 4c) but also in plasma (Figure 5c), causing a reduction of 8.0 log_10_ PFU/mL after 15 and 270 min of white light irradiation, respectively.

Although Tetra-Py(+)-Me and Tetra-S-Py(+)-Me are both symmetric porphyrins with four positive charges, their effectiveness to photoinactivate the T4-like phage was significantly different. While the singlet oxygen (^1^O_2_) production is one of the important factors to take into account in photoinactivation of pathogenic microorganisms [43,44,45,46], the Tetra-S-Py(+)-Me produces less ^1^O_2_ than Tetra-Py(+)-Me [47,48]. So, the highest efficiency of Tetra-S-Py(+)-Me in PBS and in blood plasma is probably associated to its structure and its close interaction with the viral particle and not so much with its efficacy in producing ^1^O_2_. The two tetracationic PSs have different peripheral substituents decorating the macrocycle core, resulting in different PS physicochemical properties. In Tetra-Py(+)-Me, the cationic methylpyridinium units are directly linked to the *meso* positions of the macrocycle core, whereas in Tetra-S-Py(+)-Me, there is a spacer between the porphyrin core and the less rigid methylpyridinium-4-sulphanyl subunit [48]. The higher efficacy of viral inactivation by the Tetra-S-Py(+)-Me in relation to Tetra-Py(+)-Me, mainly in plasma, can be due to a higher rotational mobility in Tetra-S-Py(+)-Me of the cationic unit, allowing a better targeted adhesion to the phage particles.

The increase in the number of positive charges borne by porphyrins may also increase the aPDT efficiency [18,41]. However, the Tri-Py(+)-Me (bearing three positive charges) was demonstrated to be significantly more effective in the T4-like phage photoinactivation in PBS and plasma than Tetra-Py(+)-Me (with four positive charges). The higher efficacy of the Tri-Py(+)-Me when compared to Tetra-Py(+)-Me is in accordance with previous results from the group [18,41]. This may be due to the asymmetric charge distribution at the periphery of Tri-Py(+)-Me, which increases the amphiphilic character of porphyrin and, consequently, improves its affinity for microorganism and viral particle targets. In fact, asymmetric PSs are significantly more effective than symmetric PSs [49,50], such as Tetra-Py(+)-Me [18,47]. Nevertheless, the symmetric porphyrin Tetra-S-Py(+)-Me (with four positive charges) proved to be significantly more effective in T4-like phage photoinactivation in PBS and plasma than Tri-Py(+)-Me. Considering that Tetra-S-Py(+)-Me produces less ^1^O_2_ than Tri-Py(+)-Me [47,48] but displays higher photodynamic efficiency in complex samples, such as plasma, beside the positive charges number and PS symmetry, the higher mobility of the PS peripheral substituent groups seems to be an important factor to take into account in the PS selection [48]. Additionally, it is noteworthy that recently MD simulations revealed that the negative electrostatic charges on the surface of the virus membrane can affect the interaction performance with cationic PSs [51,52]. In this work, it was reported that the PS binding site occurred at the connection of the S-protein stalk and head.

As opposed to the T4-like phage inactivation in PBS, in plasma the efficiency of porphyrins for anti-viral photodynamic inactivation is remarkable when compared to the MB efficacy, which is already approved for plasma disinfection. The T4-like phage inactivation with MB reached the detection limit of the method (reduction of 8 log_10_ PFU/mL) after 30 min of irradiation in PBS (Figure 4d) and did not go beyond 3 log_10_ PFU/mL of T4-like phage inactivation in plasma even after 270 min of irradiation (Figure 5d). Although the MB efficiency in the inactivation of microorganisms is highly dependent on the pH of the solution, PBS (pH 7.4) and plasma (pH 7.35 to 7.45) [53] have a similar pH, which indicates that other factors can be responsible for the high difference of aPDT’s effectiveness between PBS and plasma. The high organic content of plasma can explain the significantly lower T4-like phage inactivation with MB in plasma but also the MB photobleaching due to the high irradiance used (150 mW·cm^−2^) in the photoinactivation plasma assays. In fact, after 30 min of treatment, the T4-like phage concentration remains constant, which seems to indicate that no more MB is available to produce ROS responsible for the T4-like phage inactivation. It can be observed that the sample is colourless after 30 min of irradiation. Therefore, cationic porphyrins, mainly Tetra-S-Py(+)-Me, can be promising PSs for future clinical application in the inactivation of viruses in blood plasma.

**Figure 6:**
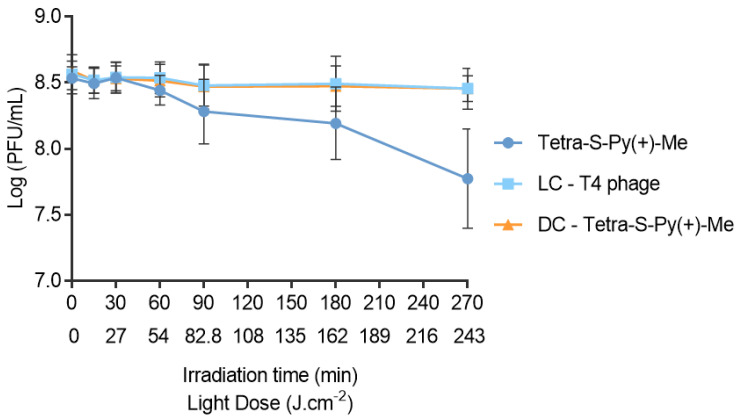
T4-like phage photoinactivation in whole blood in the presence of Tetra-S-Py(+)-Me at 10 µM and under white light irradiation at an irradiance of 150 mW·cm^−2^. All values represent the average of three independent assays in duplicate. Error bars indicate the standard deviation. Lines were used to connect the data points. LC—Light control; DC—Dark control.

Since Tetra-S-Py(+)-Me was the most effective PS in plasma, this derivative was selected also for the photoinactivation of T4-like phage in whole blood samples at the same PS concentration (10 µM), using white light at an irradiance of 150 mW·cm^−2^. The obtained results demonstrate significant differences when compared to PBS and plasma assays (Figure 6). Although Tetra-S-Py(+)-Me inactivated the T4-like phage up to the detection limit of the method in PBS and in plasma assays, in whole blood the decrease in the bacteriophage concentration did not go beyond of 1 log_10_ PFU/mL (ANOVA, *p* < 0.05), even after 270 min irradiation. In this case, light and dark controls proved that the viability of the T4-like phage was not affected by light and by PS in the dark (ANOVA, *p* > 0.05), as was verified in PBS and plasma assays. However, when the protocol was extended to MB at 10 µM, no T4-like phage inactivation was observed in the whole blood, even after 270 min.

Identical studies using porphyrins as PSs to inactivate other microorganisms also showed lower inactivation rates in whole blood [31,32,54]. This can be due not only to the large amount of organic matter present in whole blood but also to the different location of microorganisms in matrices. In plasma, the pathogens may only be in suspension, whereas in whole blood they can also be associated with cells [1,31,32] and thus may hinder the PS action. Moreover, another reason may be based on the action of haemoglobin. In fact, it is possible that the *heme* group (the iron(II) complex of protoporphyrin IX) present in haemoglobin and responsible for oxygen transport acts as an oxygen scavenger, and, consequently, less dioxygen is available to generate the required ROS for an efficient photodynamic action [22,55]. Given this fact, the effectiveness of the PSs can be affected and consequently reduced. Furthermore, haemoglobin absorbs light comprising a large fraction of the visible light, and this can also contribute to reducing significantly the number of photons available to be absorbed by the PS and, consequently, reduce the PS efficacy in whole blood [53].

The time required for significant viral inactivation by aPDT with porphyrins in the whole blood, and even in plasma, is still too long. However, porphyrin derivatives were stable dyes under all periods of irradiation, and the viral inactivation was gradual until the end of the experiments, in contrast with MB. In fact, the maximum T4-phage inactivation with MB was achieved after 15 min of irradiation (a decrease of 3 log_10_ PFU/mL) and no further reduction was detected at the end of the irradiation period (270 min). With the porphyrinic PSs we had achieved higher rates of inactivation during the period of irradiation. Consequently, improvements in the aPDT protocol using porphyrins are still needed to decrease the time required to inactivate the viruses. Improvements relative to light dose and light source may be considered and, as well, the use of chlorins or bacteriochlorins with high absorptions in the red region of the visible electromagnetic spectrum, where the light penetration is higher and the absorption by the endogenous chromophores is minimized.

## 4. Materials and Methods

### 4.1. Blood Samples

Human blood samples were voluntarily provided by Avelab clinical laboratory (Aveiro, Portugal). The samples consisted in blood test tubes containing whole blood and anticoagulant EDTA-k3 (Ethylenediamine tetraacetic acid-k3), 5.4 mg, with a final volume of 3.0 mL (BD Vacutainer^®^, Becton Dickinson, Plymouth, UK), which were used within a period of up to 5 days after harvest. The whole blood samples were centrifuged (Heraeus Megafuge 16R Centrifuge, Thermo Scientific, Waltham, MA, USA) at 2195× *g* for 5 min to obtain plasma and erythrocytes. The plasma was then separated from red blood cells and used in plasma aPDT assays. For whole blood assays, the centrifugation step was not required.

### 4.2. Bacterial Strain and Growth Conditions

The bacterial strain *Escherichia coli* ATCC 13706 was used as a T4-like phage host. The bacterial culture was stored in Tryptic Soy Agar (TSA) at 4 °C. Before each assay, some isolated colonies were transferred to 30 mL of Tryptic Soy Broth (TSB) and grown overnight for about 18 h at 37 °C with stirring (about 120 rotations per minute (rpm)). Subsequently, 0.3 mL of fresh culture was transferred to 30 mL of fresh TSB and incubated overnight under the same conditions to reach the stationary phase, corresponding approximately to a concentration of 10^8^–10^9^ colony-forming units per mL (CFU/mL) (which is equivalent to an optical density of 0.8 at 600 nm).

### 4.3. Phage Stock Preparation and Quantification

The bacteriophage of *Escherichia coli* C (ATCC 13706) (T4-like phage) was isolated from wastewater samples of the sewage network of Aveiro, and the results were previously reported by us [41]. For phage stock preparation, a morphologically representative phage plate was isolated and then added to an *E. coli* culture. This mixture was incubated at 37 °C for ~5 h with slow agitation. Subsequently, the suspension was centrifuged (Heraeus Megafuge 16R Centrifuge, Thermo Scientific) at 13,000× *g* for 10 min to remove non-infected bacteria and bacterial cell residues. The supernatant with the phage particles was kept at 4 °C after addition of 1% chloroform to prevent the growth of bacteria [41].

The quantification of bacteriophages was determined, in duplicate, by the agar double layer technique [41]. A serial dilution (1/100) of the phage suspension in PBS was prepared. Subsequently, 0.2 mL of *E. coli* bacterial host and 0.5 mL of serially diluted samples were added to a tube with 5 mL of soft TSA growth medium. The contents of the tube were mixed and then immediately poured into a Petri plate with a previously prepared confluent TSA monolayer. The plates were incubated upside down at 37 °C in the dark for ~18 h. After incubation, the number of lysis plaques was counted at the most convenient dilution (with around 30–300 lyses plaques per plate), and the number of plaque-forming units per mL (PFU/mL) was calculated. The phage titer of T4-like phage was ~10^9^ PFU/mL.

### 4.4. Photosensitizers Stock Solutions

The PSs 5,10,15-tris(1-methylpyridinium-4-yl)-20-(pentafluorophenyl)porphyrin tri-iodide (Tri-Py(+)-Me), 5,10,15,20-tetrakis(1-methylpyridinium-4-yl)porphyrin tetra-iodide (Tetra-Py(+)-Me), and 5,10,15,20-tetrakis(2,3,5,6-tetrafluoro-4-(1-methylpyridinium-4-ylsulfanyl)phenyl)porphyrin tetra-iodide (Tetra-S-Py(+)-Me) were synthetized according to the literature and their purity was confirmed by ^1^H and ^19^F NMR spectroscopy (see supplementary material, Figures S1–S5) and mass spectrometry [47,48]. The MB was purchased from Acros Organics and was used as received.

Stock solutions of Tri-Py(+)-Me, Tetra-Py(+)-Me, and Tetra-S-Py(+)-Me were prepared at 500 μM in dimethyl sulfoxide (DMSO) and stored in the dark. MB was prepared at 500 μM in PBS and also stored in the dark. All of these stock solutions were sonicated (ultrasonic cleaner, Nahita 0.6 L, 40 kHz) for 15 min at room temperature before each experiment. For the biological assays, a specific volume of the PS stock solutions was used to reach the selected final concentrations.

### 4.5. Irradiation Conditions

The photodynamic effect of the selected PSs was evaluated in PBS by exposing the samples and controls to white light provided by a LED projector (EL^®^MARK, power, voltage, and frequency of 20 W, ~230 V, and ~50 Hz, respectively) at an irradiance of 25 mW·cm^−2^, for a maximum irradiation period of 60 min.

**Figure 7:**
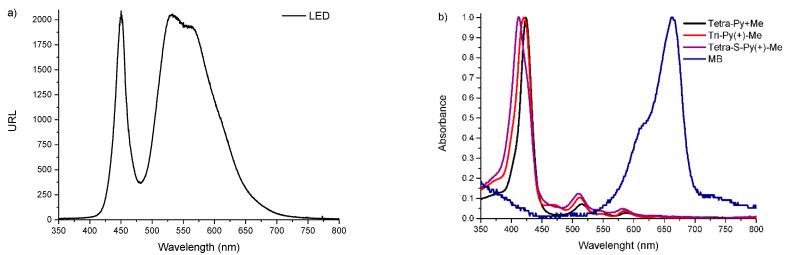
(**a**) LED emission spectra and (**b**) normalized UV-Vis absorption spectra of all PSs.

The photodynamic effect of the PSs in plasma and whole blood was analyzed by exposing the samples and controls to white light (400–800 nm) supplied from a compatible fiber optic probe attached to a 250 W quartz/halogen lamp (LUMACARE model 122, USA) with an irradiance of 150 mW·cm^−2^, for a maximum irradiation period of 270 min. The light source emission and photosensitizers absorption spectra are depicted in Figure 7.

All the irradiances were measured with a Power Meter Coherent FieldMaxII-Top combined with a Coherent PowerSens PS19Q energy sensor.

### 4.6. Evaluation of aPDT Effect on Erythrocyte Osmotic Fragility

The effect of aPDT on erythrocyte osmotic fragility was evaluated using 5.0, 10, and 20 μM of Tetra-Py(+)-Me and Tetra-S-Py(+)-Me according to the procedure reported in [31,32] and was evaluated before (incubation time) and after aPDT treatment. Initially, the PS was added to samples and dark controls and incubated for 30 min in the dark. After that period, samples and LC were irradiated at 150 mW·cm^−2^. Aliquots of 20 μL of each sample and controls were added to Eppendorf tubes with 1980 μL of NaCl solutions (0, 0.1%, 0.3%, 0.5%, 0.7%, 0.9%) and were incubated at 25 °C for 30 min with constant stirring. Finally, all samples were centrifuged at 3500 rpm for 10 min (Gyrozen 1730R, Gimpo, Korea) and the supernatants were collected. The optical density of the supernatant was determined spectrophotometrically (Multiskan FC, Thermo Scientific, Waltham, MA, USA) at 540 nm, the wavelength recommended for evaluating the amount of haemoglobin in solution. Hemolysis was represented in percentage by considering the optical density value of the distilled water solution (0% NaCl) as 100%.

### 4.7. Photodynamic Inactivation of T4-like Phage in PBS, Plasma, and Whole Blood

Before each experiment, the phage suspension was diluted in PBS (pH 7.4), plasma, or whole blood to reach 10^8^ PFU/mL. For the plasma assays, the blood samples were centrifuged as previously described. The phage suspension was equally distributed in a sterilized 12-well plate. The appropriate volume of the PS was added to achieve a final concentration of 10 µM and the suspensions were kept under magnetic stirring. During the assays, light and dark controls were included. In the light control, the phage suspension in the absence of PS was exposed to light, whereas in the dark control, the sample (phage suspension + PS) was protected from light. Then, the samples and controls were protected with aluminium foil and incubated for 30 min (T_0_) in the dark under stirring (~100 rpm) to promote the PS binding to phage particles. Afterward, the samples and controls were irradiated with white light and, depending on the experiment, with an irradiance of 25 mW·cm^−2^ for PBS assays and 150 mW·cm^−2^ for plasma and whole blood assays. As was previously mentioned, the dark control was protected from light with aluminium foil. During the experiments, the samples and controls were kept under stirring and the temperature was controlled and maintained at 25 °C.

The photodynamic effect was evaluated through phage quantification, in duplicate, by the agar double layer technique [56]. For this purpose, an aliquot of sample and controls was collected in the established times of light exposure (0, 15, 30, and 60 min for PBS assays; 0, 15, 30, 60, 90, 180, and 270 min for plasma and whole blood assays) and then serially diluted in PBS. Subsequently, 0.2 mL of bacterial host *(E. coli* overnight culture) was added to a tube with 5 mL of soft TSA growth medium. The contents of the tube were mixed and then immediately poured into a Petri plate with a previously prepared confluent TSA monolayer. After the plate dried for a few seconds, plating was performed by the drop method. The Petri plates were kept in the dark following plating for about 18 h at 37 °C. After incubation, the number of phage plaques was counted in the most convenient series of dilution and then the number of PFU/mL was calculated. At least three independent assays to each condition were done.

### 4.8. Statistical Analysis

The statistical analysis was performed with GraphPad Prism 7.04. Normal distributions were checked by the Kolmogorov—Smirnov test and homogeneity of variances was assessed by Levene’s test. Ordinary two-way ANOVA and Tukey’s multiple comparison tests were applied to assess the significance of the differences between the tested conditions. The significance of the differences verified for phage concentration was evaluated by comparing the results of each experimental treatment with that of control samples. A value of *p* < 0.05 was considered significant. Three independent assays, with two replicates, were done for each condition.

## 5. Conclusions

In conclusion, the effective photoinactivation of the T4-like phage, a mammalian virus model, with cationic porphyrins in plasma indicates that this class of compounds can be successfully used in plasma decontamination procedures mediated by aPDT. The three porphyrins—namely, Tetra-S-Py(+)-Me—can be promising PSs for virus inactivation in plasma, allowing a higher virus inactivation rate than the already approved MB. Moreover, these porphyrins did not show any significant negative effects on the erythrocytes in isotonic conditions when haemolysis was evaluated in whole blood after the aPDT protocol. The established protocol is not so effective for whole blood disinfection due to the aPDT blocking effects caused by the complexity of the matrix and by the partial filtration of the light irradiation available to the photodynamic process. Consequently, improvements in the aPDT protocol with porphyrinic derivatives are still required in order to decrease the time required to achieve an effective viral inactivation.

## Data Availability

Not applicable.

## Supplementary Materials

The following supporting information can be downloaded at: https://www.mdpi.com/article/10.3390/ijms231911548/s1.
