# Supplementary material for: Anti-Viral Photodynamic Inactivation of T4-like Bacteriophage as a Mammalian Virus Model in Blood
Source: Int J Mol Sci. 2022 Sep 30;23(19):11548. doi: 10.3390/ijms231911548 (PMC9570132; doi:10.3390/ijms231911548)
Supplement: Supplementary file 1 [file ijms-23-11548-s001.zip › ijms-1935563-supplementary.pdf]

Supporting information for

Anti-Viral Photodynamic Inactivation of T4-like Bacteriophage as a Mammalian Virus Model in Blood

Patrícia Santos [1], Ana T. P. C. Gomes [1], Leandro M. O. Lourenço [2,*], Maria A. F. Faustino [2], Maria G. P. M. S. Neves [2] and Adelaide Almeida [1,*]

[1] CESAM, Department of Biology, University of Aveiro, 3810-193 Aveiro, Portugal
[2] LAQV–REQUIMTE, Department of Chemistry, University of Aveiro, 3810-193 Aveiro, Portugal
* Correspondence: leandrolourenco@ua.pt (L.M.O.L.); aalmeida@ua.pt (A.A.)

Contents

1. NMR spectroscopy for all compounds..2

1. NMR spectroscopy for all compounds

Tri-Py(+)-Me

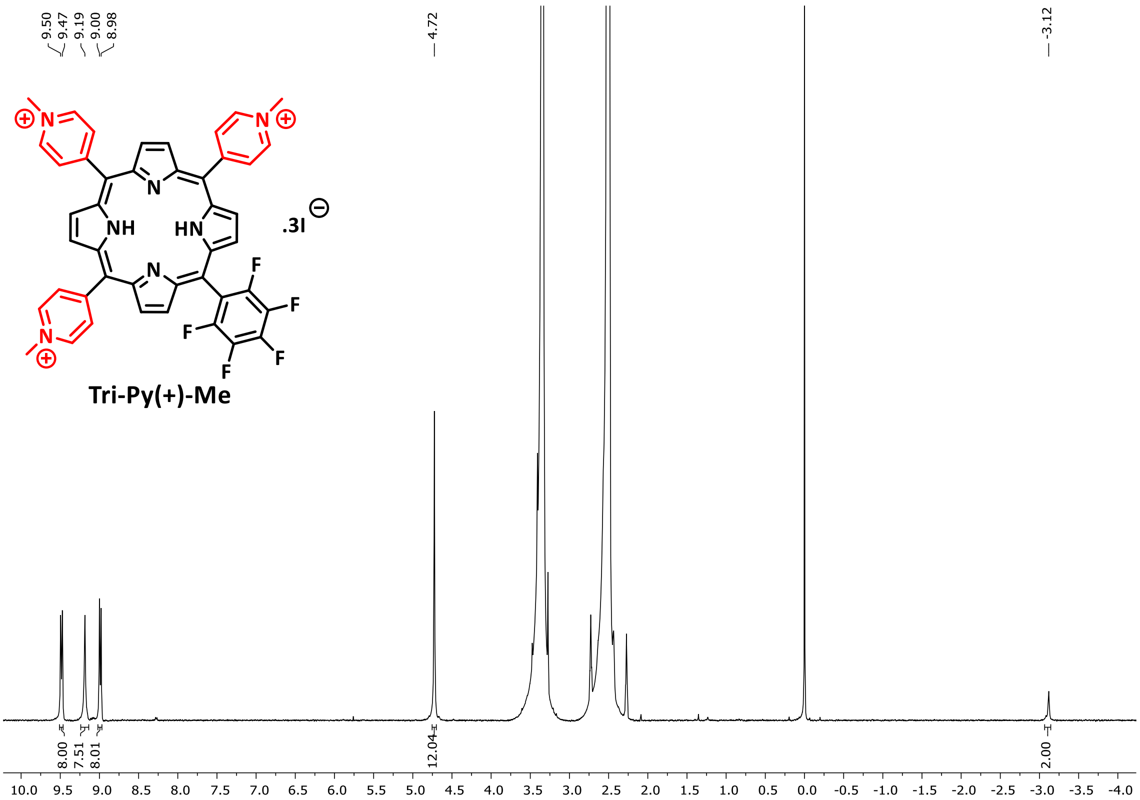

Figure S1. Spectrum of ¹H NMR of **Tri-Py(+)-Me** in deuterated DMSO-d_6. The non-integrated peaks are from solvents or internal reference signals. ¹H NMR (300.13 MHz, DMSO-d_6): δ -3.12 (s, 2H, NH), 4.72 (s, 12H, CH_3), 8.99 (d, J = 6.7 Hz, 6H, Py-*o*-H), 9.19 (s, 8H, β-H), 9.48 (d, J = 6.7 Hz, 6H, Py-*m*-H) ppm.

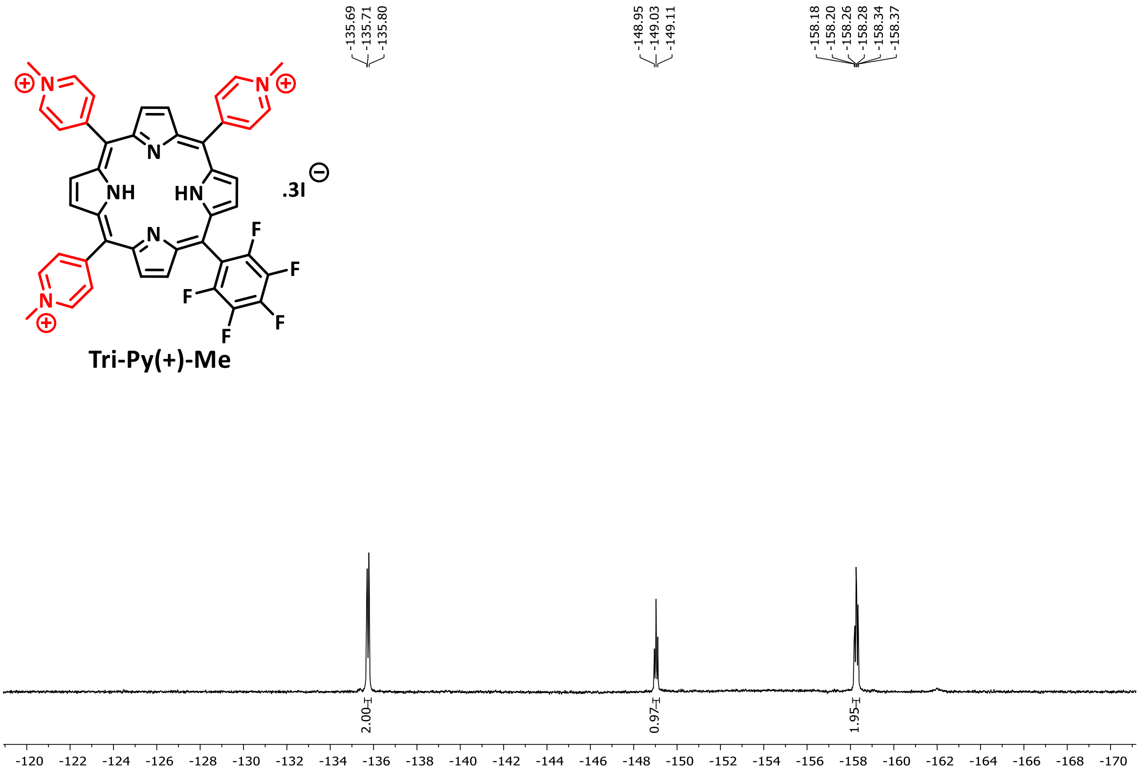

Figure S2. Spectrum of ¹⁹F NMR of **Tri-Py(+)-Me** in deuterated DMSO-d_6. The non-integrated peaks are from solvents or internal reference signals. ¹⁹F NMR (282.38 MHz, DMSO-d_6): δ -158.27 (td, J = 24.6, 6.4 Hz, 2F, *o*-F), -149.03 (t, J = 22.4 Hz, 1F, *p*-F), -135.74 (dd, J = 24.6, 6.4 Hz, 2F, *m*-F) ppm.

Tetra-Py(+)-Me

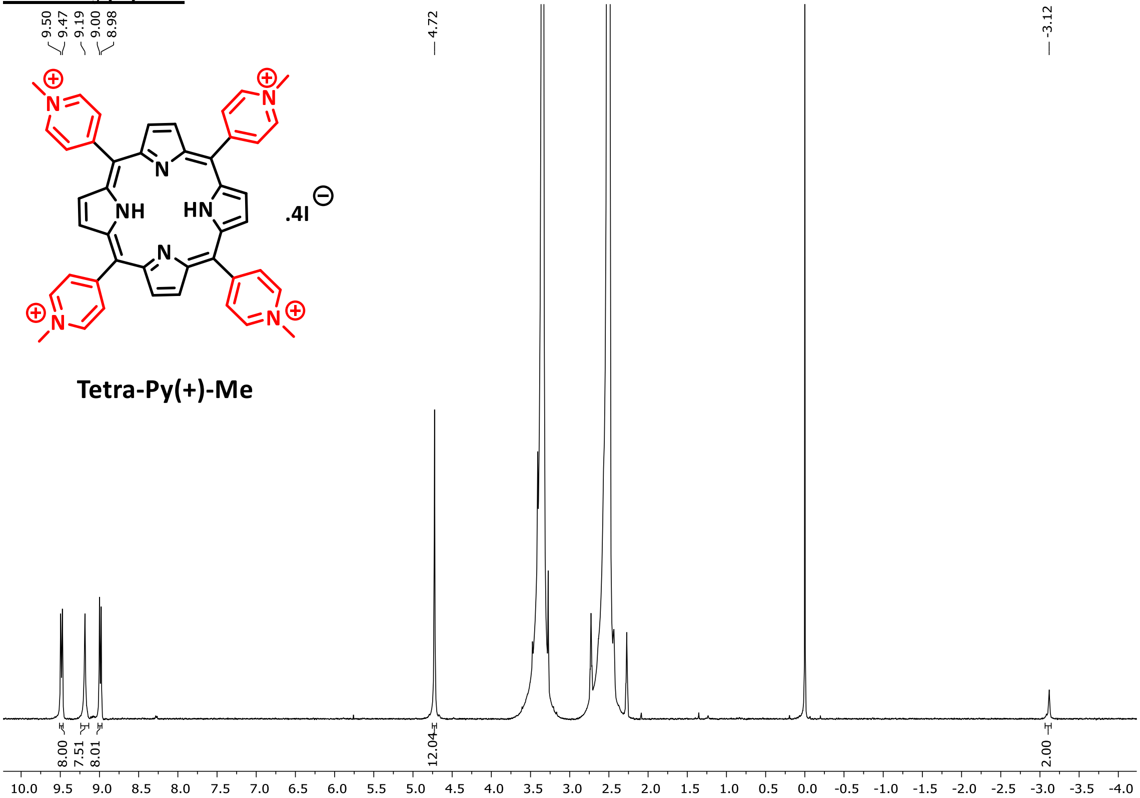

Figure S3. Spectrum of ^{1}H NMR of **Tetra-Py(+)-Me** in deuterated DMSO. The non-integrated peaks are from solvents or internal reference signals. ^{1}H NMR (300.13 MHz, DMSO-d_6): δ -3.12 (s, 2H, NH), 4.72 (s, 12H, CH_3), 8.99 (d, J = 6.7 Hz, 6H, Py-*o*-H), 9.19 (s, 8H, β-H), 9.49 (d, J = 6.7 Hz, 6H, Py-*m*-H) ppm.

Tetra-S-Py(+)-Me

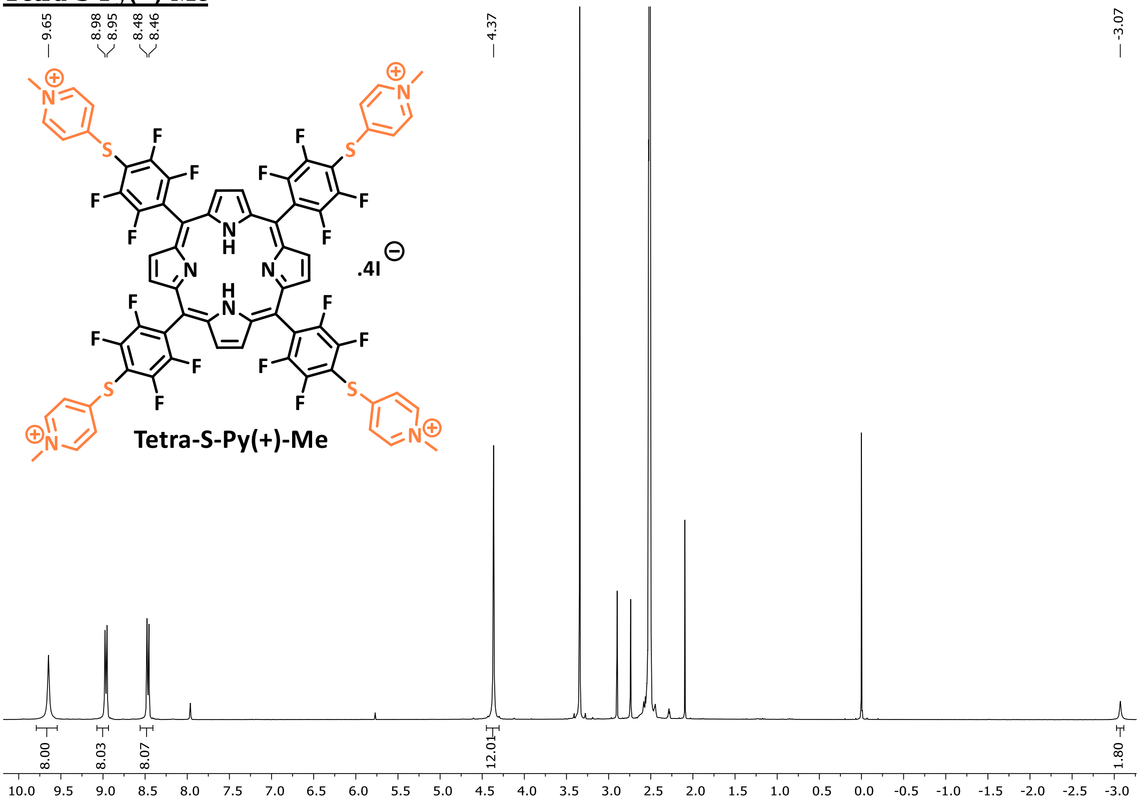

Figure S4. Spectrum of ^{1}H NMR of **Tetra-S-Py(+)-Me** in deuterated DMSO. The non-integrated peaks are from solvents or internal reference signals. ^{1}H NMR (300.13 MHz, DMSO-d_6): δ -3.07 (s, 2H, NH),

4.37 (s, 12H, CH_3), 8.47 (d, *J* = 7.1 Hz, 8H, Py-*o*-H), 8.96 (d, *J* = 7.1 Hz, 8H, Py-*m*-H), 9.65 (s, 8H, β-H) ppm.

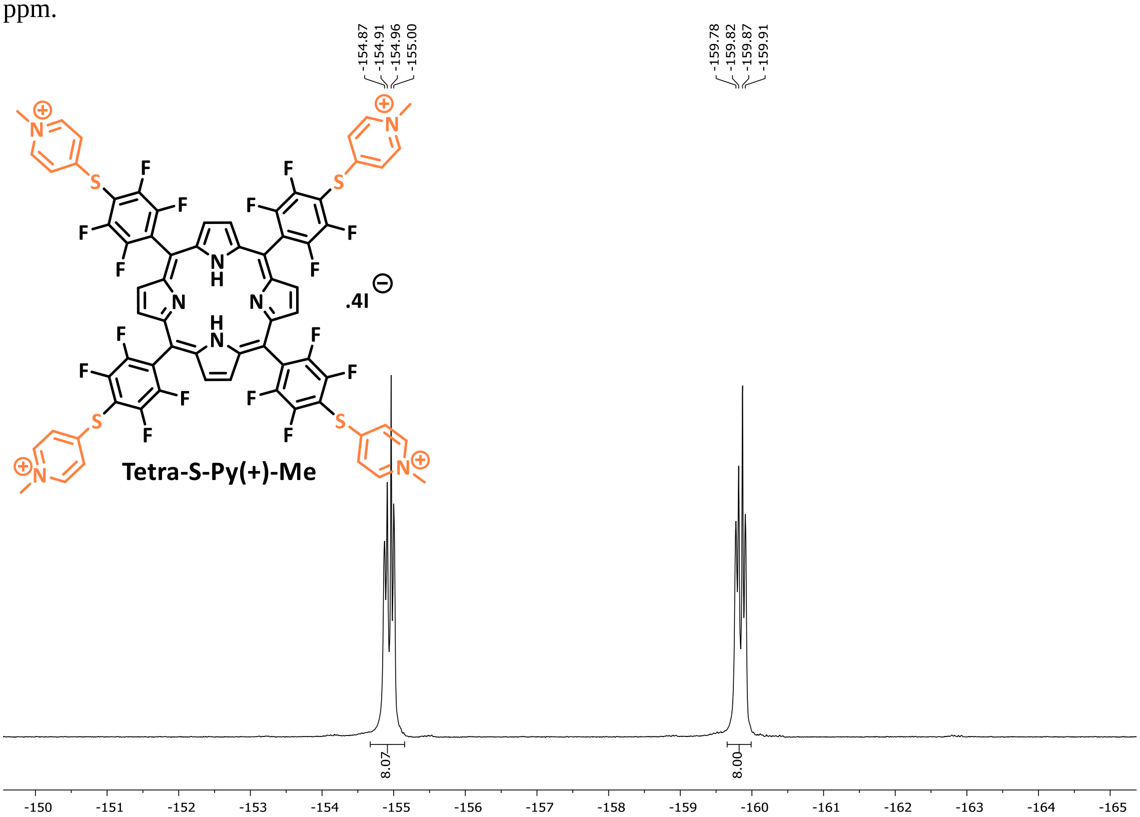

Figure S5. Spectrum of ^{19}F NMR of **Tetra-S-Py(+)-Me** in DMSO-d_6. ^{19}F NMR (282.38 MHz, DMSO-d_6): δ -159.85 (dd, *J* = 9.9 and 28.2 Hz, 8F, Ar-*o*-F), -154.94 (dd, *J* = 9.9 and 28.2 Hz, 8F, Ar-*m*-F) ppm.
